# Survival and aerobic performance of the northern shrimp are threatened by exposure to combined ocean global change drivers

**DOI:** 10.1093/conphys/coaf076

**Published:** 2025-12-24

**Authors:** Ella Guscelli, Denis Chabot, Fanny Noisette, Pierre U Blier, Mathilde Chemel, Piero Calosi

**Affiliations:** Laboratoire de Physiologie Écologique et Évolutive Marine, Département de Biologie, Chimie et Géographie, Université du Québec à Rimouski, 300 allée des Ursulines, Rimouski, QC G5L 3A1, Canada; Institut Maurice-Lamontagne, Fisheries and Oceans Canada, 850 Rte de la Mer, Mont-Joli, QC G5H 3Z4, Canada; Institut des sciences de la mer, Université du Québec à Rimouski, 300 allée des Ursulines, Rimouski, QC G5L 3A1, Canada; Laboratoire de physiologie intégrative et évolutive, Département de Biologie, Chimie et Géographie, Université du Québec à Rimouski, 300 allée des Ursulines, Rimouski, QC G5L 3A1, Canada; Laboratoire de Physiologie Écologique et Évolutive Marine, Département de Biologie, Chimie et Géographie, Université du Québec à Rimouski, 300 allée des Ursulines, Rimouski, QC G5L 3A1, Canada; Laboratoire de Physiologie Écologique et Évolutive Marine, Département de Biologie, Chimie et Géographie, Université du Québec à Rimouski, 300 allée des Ursulines, Rimouski, QC G5L 3A1, Canada

**Keywords:** Climate change, crustacean, ecophysiology, environmental impact, fisheries, integrative approach, metabolic rates, multi-driver, seafood

## Abstract

Environmental changes can influence species development, growth, size, distribution, and abundance, and when having a negative impact, they can potentially lead to a species’ decline, and ultimately its local extinction. Consequently, evaluating the impacts of ocean global change drivers, in isolation and in combination, is particularly relevant for ecologically and economically important species which guarantee food security and income for coastal communities. This study aimed to determine the physiological responses of the northern shrimp *Pandalus borealis* to different combinations of ocean warming (OW), acidification (OA) and hypoxia at multiple levels of its biological organization (i.e. from the whole-organism to the cell), to help in predicting with greater accuracy the fate of this species in a rapidly changing ocean. To do so, shrimp were exposed for 30 d to different combinations of seawater temperature (2, 6 or 10°C), pH (7.75 or 7.40 pH_T_) and oxygen (100 or 35% relative to air saturation), and their survival, whole-organism aerobic performance, and cellular energetic capacity were characterized. Our results show that shrimp were overall tolerant to the isolated effects of OW, OA and hypoxia, but when exposed to combined drivers their survival and whole-organism aerobic performance severely decreased. Isolated and combined drivers had overall no effect on enzyme activity, suggesting a low capacity for metabolic reorganization. Nonetheless, under combined drivers, we observed an adjustment of the mitochondrial enzyme stoichiometry that might help cells to maintain their energy production efficiency. Overall, the northern shrimp’s physiological status is compromised under combined ocean global change drivers, which together with the high mortality levels observed, point to a potential risk for local commercial collapse. Our results will be useful to refine mechanistic modelling for future abundance and distribution, in order to improve stock assessments, management and conservation of the northern shrimp under ongoing global changes*.*

## Introduction

The global extinction of a species results from a series of local and regional extinction events, often announced by drastic declines in abundances (Pitcher, 2001; Dulvy *et al.,* 2003), which in turn are the first alarm signals to improve the conservation and management of a species (Pitcher, 2001). Exploitation and habitat loss are currently the two major causes of local and regional extinctions in the ocean; however, global changes are expected to gain in magnitude in the near future, therefore also representing a threat to global biodiversity (Dulvy *et al.,* 2004). In fact, ocean global changes influence species’ performance, survival, productivity, and ultimately their abundance and distribution (Parmesan, 2006), and may lead to their decline and eventually extinction (Thomas *et al.,* 2004; Calosi *et al.,* 2019). To anticipate mortality level, and thus prevent species extinction, there is an urgent need to acquire an in-depth understanding of an organism’s biological and physiological response to global changes (Wikelski and Cooke, 2006; Cooke *et al.,* 2013).

The effects of global change drivers are highly dependent on the level of biological organization considered (Harvey *et al.,* 2014), with lower biological levels (e.g. cellular) defining the mechanisms underpinning the higher level of response (e.g. whole-organism), which are linked to the ecology of a species (Bartholomew, 1964). In general terms, global change effects manifest at lowers levels of biological organization, which are more sensitive, before whole-organism’s phenotypic effects are observed (Noisette *et al.,* 2021). Therefore, it is necessary to adopt an integrative approach to disentangle the complexity of biological systems, particularly within the context of the undergoing rapid environmental changes, and to investigate different levels of biological organization (Bartholomew, 1964; Harvey *et al.,* 2014).

Marine species are often considered to be less vulnerable to extinction compared to terrestrial ones, as they usually possess broad geographic ranges, are highly fecund and have a higher ability to evade capture (Roberts and Hawkins, 1999; Pinsky *et al.,* 2019). However, local and regional extinction risks should not be underestimated, especially for keystone species and/or species of high economic and food security value (Dulvy *et al.,* 2003). Indeed, the loss of valuable species might impact the global seafood market, which was valued at $159 billion in 2019 and is predicted to increase by approximately 22% by 2027 (Businesswire, 2020), as a result of increasing global human food consumption (FAO, 2018). In addition, seafood is an important source of various nutrients, such as proteins, lipids, vitamins and minerals, and contains unique fatty acids that have important health benefits (Hosomi *et al.,* 2012). Moreover, coastal zones host more than half of the world’s human population, which depend heavily on seafood to compensate for the loss in terrestrial resources threatened by climate change (Bindoff *et al.,* 2019).

Species can avoid global decline and extinction through migration into more suitable habitats (Parmesan and Yohe, 2003). Many marine species already have shifted their geographic range in response to global changes (Perry *et al.,* 2005; Parmesan, 2006; Poloczanska *et al.,* 2013; Bates *et al.,* 2014). However, some species may not be able to do so, for example due to the unavailability of alternate suitable habitats. Polar and subpolar species are especially at risk of decline when compared to species from other climatic regions, as their available habitat continues to shrink due to ongoing warming, and they cannot shift northward or southward through migration (Wassmann *et al.,* 2011). In addition, polar and subpolar species are thought to possess reduced homeostatic ability when compared to species at lower latitudes (Thor *et al.,* 2018), thus being considered among the most vulnerable of marine taxa.

The northern shrimp *Pandalus borealis,* Krøyer 1838, is a cold-adapted species with a circumpolar distribution. It has a high ecological value, as it constitutes a large proportion of the diet of adult (>25 cm) redfish (*Sebastes* spp.), Greenland halibut (*Reinhardtius hippoglossoides*, Walbaum 1792) and Atlantic cod (*Gadus morhua*, Linnaeus 1758) (Dawe *et al.,* 2012; Bernier and Chabot, 2013; Ouellette-Plante *et al.,* 2020; Brown-Vuillemin *et al.,* 2022) as well as birds, seals and whales (Parsons, 2005; Tam and Bundy, 2019). In addition, it has a high commercial value, particularly in eastern Canada (DFO, 2021). As a cold-water species, the northern shrimp lives at temperatures ranging from 1 to 6°C (Shumway *et al.,* 1985; Garcia, 2007) and is sensitive to temperature increases that affect shrimp larval survival, development, growth and shrimp adult size (Brillon *et al.,* 2005; Ouellet and Chabot, 2005; Daoud *et al.,* 2010; Arnberg *et al.,* 2013). Temperature is also known to affect metabolism of both larvae (Chabot and Ouellet, 2005; Arnberg *et al.,* 2013) and adults (Daoud *et al.,* 2007, 2010;Dupont-Prinet *et al.,* 2013 ; Hall, 2017), as well as mineral content (calcium, copper, iron, potassium, magnesium, manganese, strontium and zinc concentrations) of female abdominal muscle (Chemel *et al.,* 2020). The thermal sensitivity of *P. borealis* must be investigated within the context of the ongoing phenomenon of ocean warming (OW) (IPCC, 2022). Nonetheless, ocean acidification (OA), a global change phenomenon consisting of the decrease in ocean pH and a change in seawater carbonate chemistry (i.e. increased *p*CO_2_ and [HCO_3_^−^], and decreased [CO_3_^2−^] and carbonate saturation state) (Orr *et al.,* 2005; Feely *et al.,* 2009; IPCC, 2022), is also known to affect northern shrimp, increasing the duration of the larval development (Bechmann *et al.,* 2011; Arnberg *et al.,* 2013) and enhancing extracellular acidosis in adult males (Hammer and Pedersen, 2013). Interestingly, adults of *P. borealis* show the ability to partially compensate for extracellular acidosis, suggesting males are tolerant to increased *p*CO_2_/decreased pH (hereafter decreased pH) (Hammer and Pedersen, 2013). Finally, another important but highly understudied global phenomenon is the progressive de-oxygenation of the world’s oceans (IPCC, 2022). Within this context, male shrimp have been shown to be more tolerant than females when exposed to hypoxia (Dupont-Prinet *et al.,* 2013; Hall, 2017). Nonetheless, severe hypoxia (35–22% O_2_ saturation relative to air, % O_2_ sat. hereafter) decreases female shrimp’s aerobic performance (Dupont-Prinet *et al.,* 2013) and affects adult shrimp’s metabolic pathways (Pillet *et al.,* 2016).

Whilst our current understanding of the impacts of isolated ocean global change drivers (i.e. OW, OA and hypoxia) on the northern shrimp is relatively solid, only few studies have attempted to shed light on their combined effects. For example, Dupont-Prinet *et al.* (2013) showed that the tolerance of adults of *P. borealis* to hypoxia is reduced under increasing temperatures. In addition, survival rates decrease when shrimp are exposed to the combination of OW and OA and decrease further when the effect of hypoxia is superimposed (Dupont *et al.,* 2014; Chemel *et al.,* 2020). Yet, no study has been conducted on the combined effects of these three major ocean global change drivers on shrimp physiological responses, despite the fact they can naturally co-occur and influence each other. For this reason, the Estuary and Gulf of the St. Lawrence system (EGSL) represents an ideal study system to test the combined effects of ocean global change drivers on the physiological responses of northern shrimp. In the EGSL, shrimp are most abundant in deep waters, between 150 and 300 m deep (Dupont-Prinet *et al.,* 2013). Deep waters in this region reached temperatures of 5–6°C in the St. Lawrence Estuary (SLE) and 6–7°C in the Gulf (GSL) in 2020 (Galbraith *et al.,* 2021). These temperatures are ~ 0.5–1.5°C warmer than those observed during the period of 1991–2020, due to a reduction of cold water from the Labrador current, coupled with an increase of warm water from the Gulf Stream entering the GSL (Gilbert *et al.,* 2007; Galbraith *et al.,* 2021). Gulf Stream water masses are warmer and also oxygen depleted, thus their proportional increase represents the major cause of increasing temperatures and decreasing dissolved oxygen (DO) levels in the EGSL (Gilbert *et al.,* 2005, 2007; Jutras *et al.,* 2020). In deep waters of the EGSL, shrimp experience values of dissolved oxygen varying from about ~22–45% sat., with a decreasing trend (Bourdages *et al.,* 2022). Moreover, hypoxia and the respiration of deep water organisms are responsible for a decrease in seawater pH in bottom waters of this region (mean pH of 7.75, Mucci *et al.,* 2011).

The aim of the present study was to determine, under laboratory conditions, the impact of isolated and combined ocean global change drivers (OW, OA and hypoxia) on the survival, whole-organism aerobic performance and cellular energetic capacity, of northern shrimp in the EGSL. To do so, we exposed female shrimp to different combinations of seawater temperature, pH and oxygen for 30 d. Survival was determined daily over the exposure period, at the end of which we determined standard and maximum metabolic rates (SMR and MMR, respectively) using oxygen uptake (*Ṁ*_O2_) as a proxy (Norin and Clark, 2016; Chabot *et al.,* 2016a, 2016b). Shrimp aerobic scope (AS) was calculated as the difference between MMR and SMR (Fry, 1971), whilst the temperature coefficient (Q_10_) was used as a proxy for metabolic rate sensitivity to temperature change (Schmidt-Nielsen, 1990). Finally, we measured the activity of aerobic and anaerobic enzymes in shrimp abdominal muscle tissue as a proxy for shrimp cellular aerobic and anaerobic capacity. Based on our current understanding of global change single driver effects on adult shrimp survival and metabolism, we hypothesised that the exposure to combined OW, OA and hypoxia would reduce shrimp survival probability and aerobic capacity, both at the whole-organism and at the cellular level.

## Materials and methods

### Specimen collection, transport and maintenance

Adult individuals of the northern shrimp *P. borealis* were collected with a rigid-frame trawl in May 2018 off the coast of Rimouski (QC, Canada, ~48° 35’ N, 68° 35’ W, see Fig. 1 in Guscelli *et al.,* 2023), and transported to the Maurice-Lamontagne Institute (MLI) of Fisheries and Oceans Canada (Mont-Joli, QC, Canada). Shrimp were maintained in rectangular rearing tanks (1700 L) for approximately eight weeks before the beginning of the experiment, to allow them to adjust to laboratory conditions. For more detailed information about the collection, transport and maintenance of shrimp, see Chemel *et al.* (2020).

**Figure 1:**
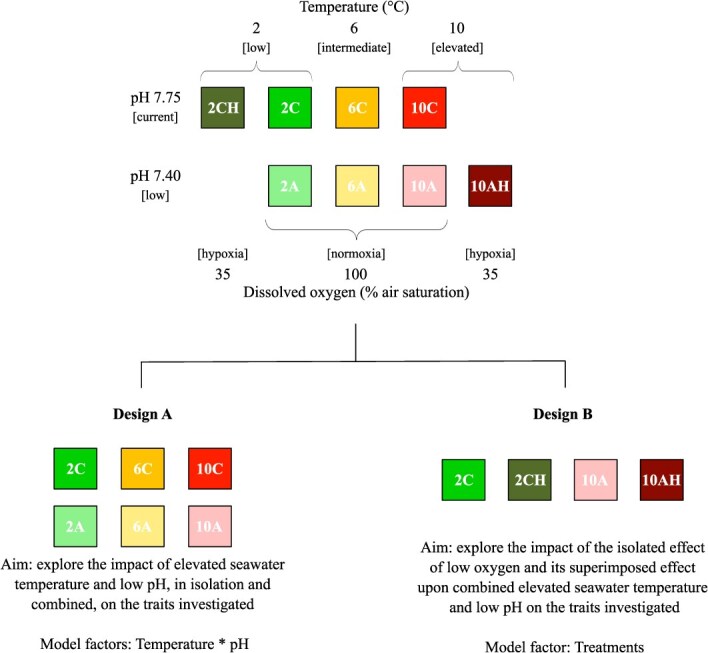
Schematic representation of the collapsed experimental design and of the two designs (Designs A and B) used to determine the effects of elevated seawater temperature, low pH and low oxygen, in isolation and combination, on the survival and physiology of female northern shrimp *P. borealis* exposed over 30 d. Treatments correspond to: 2C **(**2°C, pH 7.75, normoxia**—**green), 2A (2°C, pH 7.40, normoxia—light green), 6C (6°C, pH 7.75, normoxia—yellow), 6A (6°C, pH 7.40, nomoxia—light yellow), 10C (10°C, pH 7.75, nomoxia—red), 10A (10°C, pH 7.40, nomoxia—light red), 2CH (2°C, pH 7.75, hypoxia—dark green) and 10AH (10°C, pH 7.40, hypoxia—dark red).

### Experimental design, setup, system monitoring and protocol

The experimental design and setup were identical to those used by Chemel *et al.* (2020). To determine the effects of elevated seawater temperature, low pH and hypoxia, in isolation and in combination, on the survival as well as the whole-organism and cellular physiology of shrimp, we employed a collapsed experimental design (Fig. 1) (see Boyd *et al.,* 2018 for a definition and Britton *et al.,* 2020 for a description). Employing a collapsed experimental design allowed us to explore (i) the impact of elevated seawater temperature and low pH, in isolation and in combination, on the traits investigated (Design A) and (ii) the isolated effect of low oxygen and its superimposed effect upon combined elevated seawater temperature and low pH (Design B, treatments: 2C, 2CH, 10A, 10AH) (Fig. 1). Specifically, three levels of seawater temperature were chosen: (i) 2°C, for most shrimp populations of the northwest Atlantic, the bulk of the biomass is found at 0–4°C (see for instance, Orr and Sullivan, 2013) and 2°C was selected as a favourable temperature: an important proportion of the shrimp used for this study was found at 1–4°C in 2008–2017 (DFO, 2022); (ii) 6°C, to account for the +4°C scenario predicted globally (RCP 8.5 scenario, IPCC, 2014) and to represent recent (1990–2017) temperatures for shrimp in the Gulf of St. Lawrence (GSL) (5–6°C, DFO, 2022); and (iii) 10°C, representing conditions at the end of the century in the GSL (Lavoie *et al.,* 2020). Two levels of pH were also selected, pH 7.75 based on the current conditions in the deep waters of the EGSL (Mucci *et al.,* 2011, 2018) and pH 7.40, representing the decrease in pH between −0.3 and −0.4, predicted to occur in the EGSL by the year 2100 (RCP 8.5 scenario, IPCC, 2014). Finally, we chose two levels of dissolved oxygen (DO), normoxia 100% and hypoxia 35% air saturation, a non-lethal (chronic) level of hypoxia commonly encountered by shrimp in the EGSL (Dupont-Prinet *et al.,* 2013). Our collapsed experimental design consisted of a total of eight treatments with two replicate tanks *per* treatment, designated as follows: low temperature and current pH (2C: 2°C, pH 7.75, normoxia), low temperature and low pH (2A: 2°C, pH 7.40, normoxia), intermediate temperature and current pH (6C: 6°C, pH 7.75, normoxia), intermediate temperature and low pH (6A: 6°C, pH 7.40, normoxia), elevated temperature and current pH (10C: 10°C, pH 7.75, normoxia), elevated temperature and low pH (10A: 10°C, pH 7.40, normoxia), low temperature, current pH and low DO (2CH: 2°C, pH 7.75, hypoxia) and elevated temperature, low pH and low DO (10AH: 10°C, pH 7.40, hypoxia) (Fig. 1; for each treatment code the number indicates the temperature, the letters C and A indicate the pH level as current or acidified and the letter H indicates the hypoxic treatment).

The regulation and monitoring of temperature, pH and oxygen in the experimental setup were identical to those described in Chemel *et al.* (2020). Briefly, the open-flow experimental tanks were supplied with sea water from two reservoirs at a constant flow rate of 3.5 L min^−1^ and equipped with a regulation system. The regulation system consisted of two independent feedback systems: one that allowed the regulation of temperature (1/16 DIN Micromega autotune PID Temperature, Omega Engineering inc., Norwalk, CT, USA), and one that allowed the regulation of pH and oxygen (IKS, Aquastar, Karlsbad, Germany). The feedback system (i) regulated the automatic mixing of cold and warm water to provide each tank with sea water at the set temperature and (ii) controlled the injection of pure gaseous CO_2_ and N_2_ into each tank’s gas exchange column to maintain pH and DO levels at target values, respectively. All parameters (temperature, pH and oxygen) were independently regulated in each tank. Environmental parameters were monitored daily with handheld multimeters in each tank throughout the duration of the experiment. Carbonate chemistry parameters were calculated weekly, based on pH and alkalinity measurements, using the R package seacarb (Gattuso *et al.,* 2021). For more details about the instruments and techniques used see Chemel *et al.* (2020). Mean ± SD physico-chemical parameters for the duration of the 30 d experiment are summarized in Table 1.

**Table 1:** Summary (mean ± SD) of the physico-chemical parameters of the sea water measured and calculated (*) over the duration of the experiment (30 d) in different treatments: temperature (°C), pH (Total scale), dissolved oxygen (% sat.), salinity, total alkalinity (TA, μEq kg^−1^), total dissolved carbon dioxide* (DIC, μmol kg^−1^), carbon dioxide partial pressure* (*p*CO_2_ μatm), bicarbonate concentration* ([HCO_3_^−^], μmol kg^−1^) and carbonate concentration* ([CO_3_^2−^], μmol kg^−1^), Ω calcite* and Ω aragonite^*^

**Treatment**	**Temperature**	**pH**_**T**_	**Oxygen**	**Salinity**	**TA**	DIC *	***p*CO**_**2**_ *	**[HCO**_**3**_^**−**^ ] *	**[CO**_**3**_^**2−**^ ] *	Ω cal *	Ω ara *
2C	2.32 ± 0.10	7.76 ± 0.01	99.85 ± 0.19	32.96 ± 0.08	2071.87 ± 13.24	2065.88 ± 13.46	752.34 ± 19.79	1972.72 ± 12.98	49.56 ± 1.43	1.2 ± 0.03	0.75 ± 0.02
2A	2.35 ± 0.12	7.42 ± 0.02	99.75 ± 0.17	32.94 ± 0.08	2056.21 ± 13.07	2130.36 ± 15.09	1690.98 ± 55.31	2007.62 ± 13.65	24.29 ± 1.45	0.59 ± 0.03	0.37 ± 0.02
2CH	2.46 ± 0.11	7.74 ± 0.01	36.10 ± 1.90	32.97 ± 0.08	2067.63 ± 13.01	2065.61 ± 13.39	783.04 ± 17.39	1973.42 ± 12.83	47.09 ± 0.97	1.14 ± 0.02	0.71 ± 0.01
6C	6.02 ± 0.06	7.74 ± 0.01	98.08 ± 0.29	32.96 ± 0.07	2040.07 ± 13.33	2026.49 ± 13.74	789.28 ± 18.88	1933.12 ± 13.25	53.46 ± 1.13	1.29 ± 0.03	0.81 ± 0.02
6A	6.09 ± 0.07	7.43 ± 0.02	97.55 ± 0.15	33.07 ± 0.07	2067.63 ± 13.01	2124.2 ± 13.74	1694.84 ± 52.39	2009.68 ± 12.76	28.97 ± 1.61	0.70 ± 0.04	0.44 ± 0.02
10C	9.68 ± 0.10	7.76 ± 0.01	97.16 ± 0.20	33.11 ± 0.07	2056.21 ± 13.07	2025.04 ± 13.57	769.17 ± 21.95	1925.39 ± 13.24	65.39 ± 1.39	1.58 ± 0.03	1.00 ± 0.02
10A	9.61 ± 0.14	7.45 ± 0.02	97.26 ± 0.21	33.03 ± 0.07	2040.07 ± 13.33	2080.44 ± 15.4	1680.24 ± 68.89	1971.38 ± 14.32	34.34 ± 1.83	0.83 ± 0.04	0.52 ± 0.03
10AH	9.64 ± 0.12	7.43 ± 0.02	37.55 ± 2.04	33.18 ± 0.07	2071.87 ± 13.24	2118.49 ± 14.32	1768.75 ± 52.37	2007.71 ± 13.31	32.07 ± 1.49	0.77 ± 0.04	0.49 ± 0.02

Treatments correspond to 2C (2°C, pH 7.75, normoxia), 2A (2°C, pH 7.40, normoxia), 6C (6°C, pH 7.75, normoxia), 6A (6°C, pH 7.40, normoxia), 10C (10°C, pH 7.75, normoxia), 10A (10°C, pH 7.40, normoxia), 2CH (2°C, pH 7.75, hypoxia) and 10AH (10°C, pH 7.40, hypoxia)

For this experiment, we specifically selected non-ovigerous females to avoid any potential confounding effect linked to differences in reproductive stages and the oxygen consumption of the egg mass, but also because they are the main target of the fishery and are more sensitive than males to ocean global change drivers such as hypoxia (Dupont-Prinet *et al.,* 2013). Groups of approximately 70 females were randomly assigned to one of the 16 tanks of the experimental setup. Starting from the conditions to which shrimp were exposed to during the period of adjustment to the laboratory (4.5°C, pH 7.90, normoxia, salinity 33), the experimental tanks were gradually adjusted over 4 d until treatment values for temperature, pH and oxygen were reached, whilst always keeping salinity close to 33. The ramping period of 4 d was chosen to reduce the risk of mass mortality of shrimp exposed to the warmest treatments, particularly to the “elevated temperature, low pH and low DO treatment” for which an acute event would potentially create a bias in our results. The shrimp were then exposed for a total of 30 d to each treatment. During the exposure period the shrimp were fed *ad libitum* three times a week, with equal proportions of capelin (*Mallotus villosus*, Müller 1776) and shrimp (*Pandalus* spp.). Uneaten food was removed 24 h after each feeding period. We recorded the number of live shrimp daily, to determine survival rate throughout the experiment, and removed dead individuals and exuviae to prevent the proliferation of bacteria and ammonia accumulation. This allowed the maintenance of high water quality. The shrimp were fasted after 23 d of exposure to avoid energy demands related to digestion during the oxygen uptake measurement which started on day 28, when five individuals *per* tank (10 *per* treatment) were haphazardly selected and prepared for the measurement (see section Oxygen uptake measurement below for details). At the end of each oxygen uptake measurement, at day 30, individuals were removed from the respirometer, gently blotted with tissue paper, and weighed with a digital high-precision scale (Mf-300, A&D Company, Tokyo, Japan; ± 0.001 g) in order to determine wet mass (WM). Finally, to preserve the abdomen muscle tissue for further analyses, shrimp were rapidly dissected on ice, to prevent tissue degeneration. The procedure lasted less than 60 sec. The cephalothorax and the carapace were carefully removed using a ceramic knife and plastic forceps. The abdomen muscle tissue, representing the largest proportion of body mass, was then sectioned into three equal parts (approximately 1 g each), placed in Eppendorf tubes and flash-frozen in liquid nitrogen, to instantly interrupt all biochemical reactions. Samples were stored at −80°C for further analyses.

### Metabolic traits and temperature coefficient

In order to determine the effect of combined ocean global change drivers on shrimp metabolic rates, individuals’ oxygen uptake (*Ṁ*_O2_, in mg O_2_ h^−1^) was used as a proxy (Fry, 1971; Norin and Clark, 2016; Chabot *et al.,* 2016a, 2016b) and measured *via* intermittent-flow respirometry in static respirometers (Steffensen, 1989; Svendsen *et al.,* 2016) for all treatments. Specifically, resting condition *Ṁ*_O2_ was used as a proxy for standard metabolic rate (SMR) (Fry, 1971; Chabot *et al.,* 2016a) and post-exercise *Ṁ*_O2_ was used as a proxy for maximum metabolic rate (MMR) (Fry, 1971; Norin and Clark, 2016). We then calculated the aerobic scope (AS = MMR—SMR) (Fry, 1947) as it provides a tangible estimate of the aerobic metabolic rate available to an animal above SMR. Additionally, the temperature coefficient (Q_10_) was calculated for both SMR and MMR to assess the sensitivity of these traits to temperature variations (Schmidt-Nielsen, 1990) for both pH conditions.

#### Respirometry method and setup

Oxygen uptake was measured through intermittent-flow respirometry (Svendsen *et al.,* 2016). It consists of an alternating series of closed phases permitting the measurement of oxygen uptake via the decrease in oxygen content of the water, and open phases when water flow resumes to renew the water inside the respirometry chambers (hereafter respirometers). Respirometers were kept in two dedicated respirometry tanks, independent from the experimental tanks, but identical in their setup (see section Experimental design, setup, system monitoring and protocol above for details). Each respirometry tank was filled with sea water at the same temperature, pH and DO as the tank the shrimp were sampled from. Each respirometry tank hosted ten custom-built cylindrical glass respirometers (vol. ≈300 ml) and two submersible flush pumps (1048, Eheim, Stuttgart, Germany) with two PVC manifolds that supplied clean and aerated seawater to five respirometers each, during the open or flush phases. Respirometers were flushed regularly (see section Oxygen uptake measurement below for details) and water overflow was evacuated above the water surface to create an airlock and prevent the exchange of oxygen between respirometers and ambient water during the closed phases (Svendsen *et al.,* 2016). Each respirometer was equipped with a recirculation loop, consisting of a recirculating pump (AD20P-1230C, DollaTek, Hong Kong, China) and Tygon tubing, to ensure good mixing (Svendsen *et al.,* 2016). The schematic for a single respirometer is shown in Fig. 6a of Svendsen *et al.* (2016). During the closed phase and after a stabilization period, DO was measured every second with fibre optic oxygen probes (OXYPro DP-PSt3, PreSens Precision Sensing GmbH, Regensburg, Germany) placed on the positive pressure side of the recirculation loop and coupled to an oxygen meter (Oxy-4 mini or Fibox 3, PreSens Precision Sensing GmbH).

Data acquisition and flush activation were controlled by a data acquisition unit (DAQ-M, Loligo systems apS, Viborg, Denmark) linked to a dedicated software (AutoResp 2™, Loligo systems apS).

#### Oxygen uptake measurement

In order to measure shrimp oxygen uptake, specimens were first individually transferred to a flow-through tank (i.e. exhaustion tank) where seawater parameters matched those of the specific experimental treatment from which the shrimp was haphazardly selected. Here, individuals were chased to exhaustion, defined as the moment they were not able to flick their tail anymore. Time of exhaustion and the number of tail flicks were recorded. Immediately, the shrimp were exposed for 1 min to air to further increase their oxygen debt (method modified from Roche *et al.,* 2013), and then rapidly but carefully transferred into individual respirometers. During the first 2 h or so (long closed phase), the respirometers were not flushed, to ensure that the fastest rate of DO decline did not occur during a flush phase. For the rest of the experiment (over 2 d) respirometers were flushed with experimental treatment seawater for 300 s and closed for 660 s to enable multiple *Ṁ*_O2_ measurements used to estimate SMR. The linear decline in DO observed during the last 480 s of the closed phase was used to calculate *Ṁ*_O2_ according to Steffensen (1989; equation 2) and Garcia and Gordon (1992; equation 8) for oxygen solubility. Background *Ṁ*_O2_ was measured before shrimp were introduced into the respirometers and after shrimp removal, and a linear regression was fitted to the background respiration values. This regression of background respiration as a function of time for each shrimp was used to correct *Ṁ*_O2_.

#### Determination of SMR and MMR

Shrimp SMR and MMR were determined from the raw data acquired by the AutoResp 2™ software (DO as a function of time). To improve signal to noise ratio, raw data were first smoothed with a running average with window width varying from 15 to 75 s depending on the amount of noise present in the data for a given shrimp. The low signal to noise ratio was caused by a relatively high ratio of water volume to shrimp mass (40.6 ± 5.7). The smooth signal was then split into 20-s blocks and the median DO of each block was retained. This further improved the signal to noise ratio (Chabot *et al.,* 2021). Processed traces of DO over time were plotted and *Ṁ*_O2_ recalculated for all slopes for a given shrimp. These plots were examined to select an appropriate minimal value of *r*^2^ below which slopes were deemed unusable (too noisy or not linear). For most shrimp, minimum *r*^2^ was set to 0.90 or more (see Table S1  in  Supplementary  Material for details), but for two shrimp, *r*^2^ was decreased to 0.87 and 0.80 to include a higher proportion of slopes, which were judged to be linear despite their lower *r*^2^, to avoid underestimating SMR (Chabot *et al.,* 2021). For each shrimp, SMR was determined using the quantile method detailed in Chabot *et al.* (2016b), with a *q* of 0.2, after excluding *Ṁ*_O2_ determinations obtained during the recovery and acclimation periods: 15 and 24 h for normoxia and hypoxia treatments, respectively, as visual examination of the plots of *Ṁ*_O2_  *versus* time showed that shrimp required more time to pay their oxygen debt and acclimate to the respirometer under hypoxia conditions.

MMR was estimated as in Zhang *et al.* (2019). Oxygen uptake typically slows down rapidly when a fatigued animal is placed in a static respirometer and calculating regressions of DO over time using short time periods reduces the likelihood of underestimating MMR. The shortest reliable window width was estimated to be 3 min (Supplementary Material—Determination of the shortest reliable interval to estimate maximum metabolic rate (MMR)). Rolling regressions (i.e. regressions calculated over 3 min, each one starting 1 s later than the previous one) were calculated over the long closed phase and the steepest slope was taken to calculate MMR. A second estimate of MMR for each shrimp was the highest *Ṁ*_O2_ observed during the regular intermittent-flow cycles, due to spontaneous activity. MMR was the highest of the two values (Dupont-Prinet *et al.,* 2013) and in the majority of cases (48 of 71 shrimp kept in the final dataset; see below), it was the post-chase value.

Of the 80 shrimp for which SMR and MMR were determined via respirometry, nine individuals were removed from the final dataset: six shrimp moulted and/or died in the respirometers and three others did not reach low stable *Ṁ*_O2_ levels (they did not acclimate to the respirometer), or failed to increase *Ṁ*_O2_ following exercise.

#### Temperature coefficient calculation

The temperature coefficient (*Q*_10_) of female shrimp was calculated for SMR and MMR at both pH conditions as: *Q*_10_ = (*R*_2_/*R*_1_) ^10/(*T*2—*T*1)^

where R_1_ and R_2_ are the mean metabolic rates (mean SMR and mean MMR) at temperatures *T*2 > *T*1 (Schmidt-Nielsen, 1990).

### Cellular aerobic and anaerobic capacity

We measured aerobic and anaerobic enzyme activity in shrimp abdomen muscle tissue as a proxy for shrimp cellular energetic capacity. To relatively determine aerobic capacity, we measured the activity of citrate synthase (CS) as a proxy for mitochondrial density (Moyes *et al.,* 1997; Rabøl *et al.,* 2006), cytochrome C oxidase (COX) as a proxy for aerobic metabolic capacity (Marie *et al.,* 2006) and complexes I and III (electron transport system, ETS) as a proxy for maximum mitochondrial oxygen consumption rate (Schmidlin *et al.,* 2015). It has been proposed that the CS activity might reflect the mitochondrial volume density in a cell as CS is a key enzyme of the Krebs cycle included in the mitochondrial matrix; therefore, if the relative volume of mitochondria changes in the cell or the tissue, it is expected to observe a proportional change in CS activity (Meinild Lundby *et al.,* 2018). The COX is the last complex of the ETS and the one that catalyses the controlled reduction of molecular oxygen with electrons provided by Cytochrome C. Its activity should therefore reflect the maximal capacity of mitochondria to reduce molecular oxygen since oxidative capacity is determined by ETS.

Additionally, we measured the lactate dehydrogenase (LDH) activity, which is a proxy for anaerobic glycolytic capacity (Farhana and Lappin, 2021). We then calculated the ratio of (i) CS to ETS activities and (ii) COX to ETS activities as any divergence between CS to ETS activity and COX to ETS activities expresses differences in mitochondrial morphology or structural and functional organization. Considering that ETS is the measure of the electron flux from NADH to the last electron acceptor through Complex I, Ubiquinone pool and Complex III and that it is considered as a maximum flux limiting step (Lemieux and Blier, 2022), divergences between CS to ETS activity will inform on the relative capacity of mitochondria to reduce (provide electrons) the ETS, while divergences between COX to ETS will be indicative of the relative capacity of mitochondria to both oxidize the ETS and reduce oxygen. In this sense, for example, an increase of the relative activity of CS over ETS in a given environmental condition would suggest that steps previous to ETS were constrained under such conditions and the acclimatory response would have been to increment this capacity. This adjustment would have been obtained by modification of mitochondrial organization instead of mitochondrial content; (iii) CS to LDH activities and (iv) COX to LDH activities as any divergence between CS to LDH activity and COX to LDH activities should express differences in metabolic organization. For example, knowing that CS is located in the mitochondrial matrix and thus is a marker of mitochondrial content (Larsen *et al.,* 2012), and that LDH is a key enzyme of the anaerobic glycolysis, changes in this ratio resulting from adaptation (Hunter-Manseau *et al.,* 2019) or acclimation (Pereira *et al.,* 2018) can inform on the adjustment needed to improve either relative aerobic or anaerobic capacity; and (v) CS to COX activities as any divergence between CS to COX activity suggests differences in mitochondrial morphology. Indeed, this ratio relates to the surface to volume ratio of mitochondria: as stated above, CS is included in the mitochondrial matrix and thus linked to the volume of mitochondria, whilst COX is part of the ETS embedded in the inner mitochondrial membrane (IMM) and its content depends on the overall IMM surface density in the cell. Therefore, any change in the ratio should express modification of the relative capacity of the TCA over the reduction capacity of COX and ETS. This might result from adjustment of the shape of mitochondria that regulate mitochondrial functions (Lee *et al.,* 2003; Nisoli and Carruba, 2006; Jacobi *et al.,* 2015; Buck *et al.,* 2016; Heine *et al.,* 2023).

To measure enzyme activity, frozen abdomen muscle tissue samples were rapidly minced on ice with a razor blade (11–515 1–1/2 inch, Stanley, Towson, Maryland) and weighed with a high precision digital scale (Quintix X124-1S, Sartorius, *Göttingen*, Germany; ±0.0001 g). Samples were then homogenized on ice using a motorized homogenizer (Polytron PT 1200, Kinematica AG, Luzern, Switzerland) with a volume of phosphate buffered saline solution (PBS, pH 7.5) containing 0.1% Triton X-100 and 1 mM methylene diamine tetra-acetic acid (EDTA) that was four times the weight of the sample. Post homogenization, samples were centrifuged (500 *g*) for 5 min at 4°C (centrifuge 5810 R, Eppendorf, Hamburg, Germany). Finally, the supernatant was divided into aliquots for enzyme activity measurements. The CS, COX and LDH activities were measured according to Thibeault *et al.* (1997) and the ETS activity was measured according to Lannig *et al.* (2003). Total protein content was determined on homogenates using the bicinchoninic acid method (Smith *et al.,* 1985) with BSA as standard. For each analysis, homogenates were diluted to obtain linear reaction slopes for a minimum of 5 min. The dilution factor was 5 for CS, COX and protein content analyses, 20 for ETS and 100 for LDH. Analyses were carried out at a constant temperature of 20°C using a spectrophotometer (Cary 100 UV–Vis, Agilent, Petaling Jaya, Malaysia) and the proprietary software (CaryWin UV, 4.20). Total protein content was measured using a UV/VIS microplate spectrophotometer (Perkin Elmer Envision, Foster City, CA, USA). All chemicals used for these assays were obtained from Sigma-Aldrich or Thermo Fisher Scientific. The enzymatic activities were measured and expressed as U g^−1^ of wet tissue and U g^−1^ of protein for total and specific activity, respectively. All analyses were performed in duplicate and if the two measurements were more than 10% apart, the analysis was repeated. For seven shrimp the COX activity measurements remained more than 10% apart, thus those shrimp were removed from the dataset for the analyses on COX activity and ratios involving COX.

### Statistical analysis

In order to test for the isolated and combined effects of elevated temperature, low pH and low oxygen on shrimp survival over 30 d, survival was expressed as survival probability (i.e. the probability of surviving at the end of the 30 d exposure period) and survival probability curves were compared among treatments using a Kaplan–Meier plot. On day 28, five shrimp per tank were removed from the experimental tank for respirometry measurements. Thus, they were declared right censored. To compare survival probability curves between treatments, we first performed a log-rank test (survival package, Therneau and Lumley (2015)) to compare survival curves within treatments to determine if the two replicate tanks differed. As no significant difference between replicates was found (min *P*-value = 0.11), shrimp from replicate treatments were pooled to produce a Kaplan–Meier plot complemented by a log-rank test. Bonferroni corrected log-rank *post hoc* tests were then computed to compare the survival curves of the different treatments.

Mixed effect models (lmerTest package, Kuznetsova *et al.* (2017); and lme4 package, Bates *et al.* (2015)) were first used to test the effect of ocean global change drivers on shrimp SMR, MMR, AS and enzyme activities and ratios. The effect of ocean warming and acidification on the traits investigated was tested, in isolation and in combination, on treatments employed in Design A. Temperature and pH were set as crossed fixed factors, while tank ID was set as a random variable to account for the non-independence of shrimp in the same tank. Additionally, Design B was used to explore the isolated effect of low oxygen and its superimposed effect upon combined elevated seawater temperature and low pH on the traits investigated: treatments were set as fixed factors and tank ID as a random variable. For both designs, SMR, MMR, AS and wet mass (WM) were log_10_ transformed to meet the assumption for linearity and log_10_ WM was set as covariate to control for the effect of mass differences on metabolic rates (Schmidt-Nielsen, 1984). In addition, as log_10_ WM had a significant effect on log_10_ SMR and log_10_ AS when analyzed to test the effects of treatments, it was kept as covariate for all analyses on metabolic rates. The effect of mass on SMR, MMR and AS between treatments was verified testing the homogeneity of the slopes, which were overall considered to be homogeneous between treatments. The term “tank” was removed as it never exerted a significant effect on the variables studied. Consequently, we performed an ANCOVA on SMR, MMR and AS and an ANOVA on enzyme activity and ratios (lmTest package, Hothorn *et al.* (2015)). Tukey HSD tests (Hothorn *et al.,* 2008) were used to conduct *post hoc* analyses when significant effects were evidenced. The normality of distributions were tested using the Shapiro–Wilk’s test. COX data (in Design A), ETS data and CS:COX data were log_10_ transformed to meet the normality assumption. Homoscedasticity was verified using both the Brown–Forsythe’s and Fligner–Killeen’s tests. In all cases, the variances were homogeneous with the exception of log_10_ CS:COX, for which we performed a Welch’s ANOVA followed by Games–Howell *post hoc* tests to analyse the effect of hypoxia and its superimposed effect upon combined elevated seawater temperature and low pH on this trait (rstatix package, Kassambara (2021)). For all *post hoc* tests, the *α* was set to 0.05 and we did not report the exact results. A visual analysis of the residuals was also performed to confirm the appropriateness of the models used. Pearson’s correlations between total and specific enzymatic activity responses were verified for all enzymes. For all enzymes measured in shrimp muscle tissue, total and specific enzymatic activity responses were significantly correlated (*r* between 0.80 and 0.97 depending on the enzymes, *P* < 0.001 for all analyses) so only specific activity responses are presented. All analyses were performed using the software R 3.6.3 version (R Core Team, 2020).

## Results

### Survival

**Figure 2:**
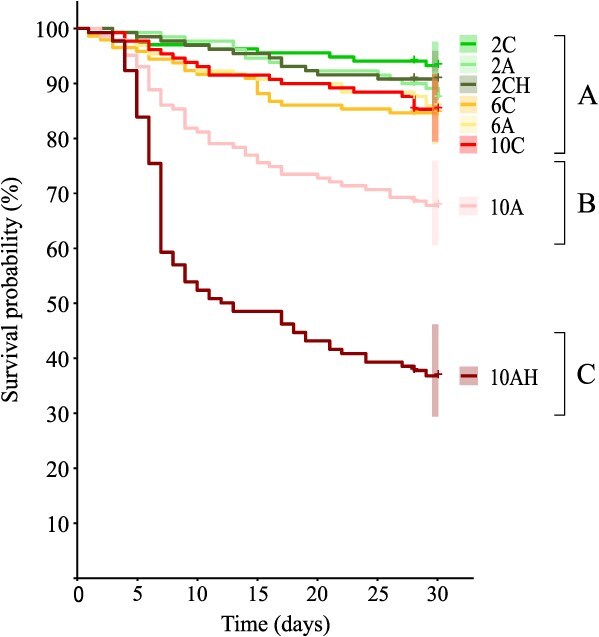
Kaplan–Meier plot of survival probability curves for female shrimp *P. borealis* exposed over 30 d to elevated temperature, low pH and low oxygen, in isolation and combined. Each curve represents the survival probability of a specific treatment, and the shaded areas at day 30 represent its 95% CI. Upper case letters identify significant differences (*P* < 0.05) among treatments.

Survival probability curves for females of the northern shrimp *P. borealis* exposed for 30 d to different combinations of ocean global change drivers are presented in Fig. 2. Differences in survival among different treatments were observed (χ^2^_7,1067_ = 256, *P* < 0.0001). In more detail, exposure to the 10AH treatment resulted in the fastest and greatest reduction in survival over the exposure period, which drastically dropped to approximately 40%, being significantly different from all other treatments. For the 10A treatment, survival probability decreased less acutely when compared to the 10AH treatment, but was still significantly different from all other treatments. Finally, survival probability curves did not significantly differ among all other treatments tested, and their average survival was 88% at the end of the 30 d exposure period.

### Standard metabolic rate

Mean SMR measured in the northern shrimp exposed to elevated temperature, low pH and low oxygen in isolation and combined are summarized in Table 2.

**Table 2:** Summary (mean ±SE) of morphological and physiological traits measured in females of the northern shrimp *P. borealis* exposed over 30 d to elevated temperature, low pH and low oxygen, in isolation and in combination

	Treatment	**2C**	**2A**	**2CH**	**6C**	**6A**	**10C**	**10A**	**10AH**
		*n* = 9	*n* = 9	*n* = 9	*n* = 9	*n* = 9	*n* = 8	*n* = 9	*n* = 9
	WM (g)	8.41 ± 0.22	9.98 ± 0.62	9.14 ± 0.50	9.33 ± 0.30	9.06 ± 0.39	9.08 ± 0.17	8.89 ± 0.39	10.16 ± 0.58
Metabolic traits	SMR (mg O_2_ h^−1^)	0.38 ± 0.03	0.36 ± 0.02	0.32 ± 0.02	0.52 ± 0.02	0.56 ± 0.06	0.61 ± 0.03	0.70 ± 0.05	0.72 ± 0.05
	MMR (mg O_2_ h^−1^)	1.66 ± 0.11	1.42 ± 0.15	0.75 ± 0.05	2.27 ± 0.17	1.86 ± 0.11	2.30 ± 0.21	2.09 ± 0.15	1.23 ± 0.05
	AS (mg O_2_ h^−1^)	1.27 ± 0.10	1.06 ± 0.15	0.44 ± 0.05	1.75 ± 0.16	1.30 ± 0.10	1.69 ± 0.24	1.39 ± 0.15	0.51 ± 0.06
Cellular energetic capacity	CS (U g^−1^ proteins)	6.405 ± 0.45	5.397 ± 0.56	5.344 ± 0.71	6.667 ± 0.71	4.610 ± 0.68	6.098 ± 0.40	5.373 ± 0.78	5.262 ± 0.50
	COX (U g^−1^ proteins)	1.931 ± 0.15	1.810 ± 0.19	2.343 ± 0.40 (*n* = 7)	2.327 ± 0.41 (*n* = 8)	2.051 ± 0.27 (*n* = 8)	2.445 ± 0.19 (*n* = 7)	1.756 ± 0.21 (*n* = 8)	2.758 ± 0.35 (*n* = 8)
	ETS (U g^−1^ proteins)	33.132 ± 2.13	30.825 ± 3.46	33.511 ± 3.38	37.326 ± 4.86	30.976 ± 1.93	36.802 ± 1.86	32.239 ± 2.55	34.709 ± 2.07
	LDH (U g^−1^ proteins)	326.842 ± 30.28	365.748 ± 37.07	375.703 ± 38.54	337.778 ± 38.76	323.130 ± 45.02	389.768 ± 33.30	320.924 ± 26.80	398.635 ± 35.98
	CS:ETS	0.198 ± 0.02	0.183 ± 0.02	0.158 ± 0.02	0.188 ± 0.02	0.148 ± 0.02	0.167 ± 0.01	0.166 ± 0.02	0.158 ± 0.02
	COX:ETS	0.061 ± 0.01	0.060 ± 0.004	0.068 ± 0.01 (*n* = 7)	0.059 ± 0.01 (*n* = 8)	0.067 ± 0.01 (*n* = 8)	0.067 ± 0.01 (*n* = 7)	0.058 ± 0.01 (*n* = 8)	0.080 ± 0.01 (*n* = 8)
	CS:LDH	0.021 ± 0.002	0.015 ± 0.001	0.015 ± 0.003	0.020 ± 0.002	0.015 ± 0.002	0.016 ± 0.002	0.017 ± 0.002	0.014 ± 0.001
	COX:LDH	0.0061 ± 0.0004	0.0052 ± 0.0007	0.0064 ± 0.0011 (*n* = 7)	0.0064 ± 0.006 (*n* = 8)	0.0062 ± 0.0007 (*n* = 8)	0.0063 ± 0.0006 (*n* = 7)	0.0060 ± 0.0009 (*n* = 8)	0.0079 ± 0013 (*n* = 8)
	CS:COX	3.37 ± 0.17	3.29 ± 0.57	2.81 ± 0.26 (*n* = 7)	3.35 ± 0.33 (*n* = 8)	2.51 ± 0.29 (*n* = 8)	2.51 ± 0.31 (*n* = 7)	3.01 ± 0.60 (*n* = 8)	1.97 ± 0.24 (*n* = 8)

Here, we provide per treatment: wet body mass (WM); metabolic traits—standard metabolic rate (SMR), maximum metabolic rate (MMR) and aerobic scope (AS); cellular energetic capacity—specific activity of citrate synthase (CS), cytochrome C oxidase (COX), electron transport system (ETS), lactate dehydrogenase (LDH), and ratios for CS—ETS (CS:ETS), COX—ETS (COX:ETS), CS—LDH (CS:LDH), COX—LDH (COX:LDH) and CS—COX (CS:COX).

**Figure 3:**
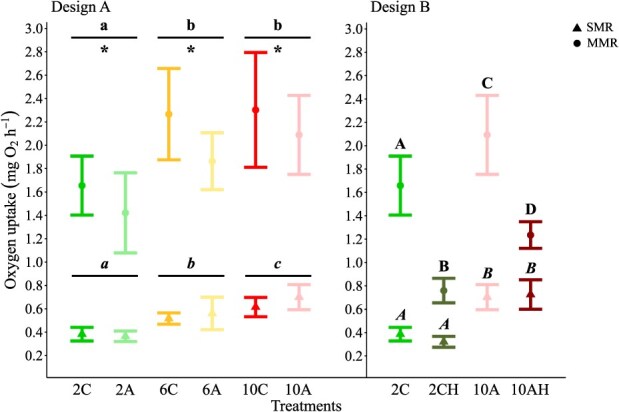
The effects of 30 d exposure to elevated temperature, low pH and low oxygen, in isolation and combination (Designs A and B), on mean SMR and MMR of female shrimp *P. borealis* are represented by triangles and dots, respectively. Triangles and dots represent the mean value and the associated error bars the 95% CI. Italic and classical lower case letters identify significant differences (*P* < 0.05) among temperature treatments independently of pH for mean SMR and MMR, respectively, whilst asterisks identify significant differences (*P* < 0.05) between pH treatments, combining all temperatures. Italic upper-case letters indicate significant differences (*P* < 0.05) among treatments for mean SMR and classical uppercase letters indicate significant differences (*P* < 0.05) among treatments for mean MMR.

Design A: Mean SMR increased significantly with increasing temperatures (Table 3, Fig. 3), along a linear trend, from 0.37 to 0.65 mg O_2_ h^−1^ at 2 and 10°C, respectively (*P* < 0.05). In addition, no effect of pH in isolation or of the interaction between temperature and pH on this trait was found (Table 3).

**Table 3:** Results for the best-fitted model testing the effect of 30 d exposure to elevated temperature, low pH and low oxygen, in isolation and in combination (Designs A and B) on the metabolic traits and cellular energetic capacity of females of the northern shrimp *P. borealis*

	Best-fitted model—Design A													Best-fitted model—Design B						
		Temperature			pH			Temperature × pH			Wet mass				Treatment			Wet mass		
		*df*	*F*	*p*	*df*	*F*	*p*	*df*	*F*	*p*	*df*	*F*	*p*		*df*	*F*	*p*	*df*	*F*	*p*
Metabolic traits	log_10_ SMR	2	44.135	**<0.001**	1	0.286	0.595	2	2.415	0.101	1	3.261	0.077	log_10_ SMR	3	43.192	**<0.001**	1	39.389	**<0.001**
	log_10_ MMR	2	13.386	**<0.001**	1	7.099	**0.011**	2	0.740	0.483	1	1.588	0.214	log_10_ MMR	3	65.621	**<0.001**	1	0.140	0.711
	log_10_ AS	2	3.552	**0.037**	1	6.182	**0.017**	2	0.354	0.704	1	0.605	0.441	log_10_ AS	3	29.172	**<0.001**	1	12.574	**0.001**
Cellular energetic capacity	CS	2	0.097	0.908	1	6.325	**0.015**	2	0.642	0.531				CS	3	0.749	0.531			
	log_10_ COX	2	0.488	0.617	1	3.276	0.077	2	0.740	0.483				COX	3	2.590	0.073			
	log_10_ ETS	2	0.521	0.598	1	3.540	0.066	2	0.052	0.950				log_10_ ETS	3	0.226	0.878			
	LDH	2	0.213	0.809	1	0.222	0.640	2	1.117	0.336				LDH	3	1.294	0.293			
	CS:ETS	2	1.106	0.339	1	1.624	0.209	2	0.596	0.555				CS:ETS	3	1.244	0.310			
	COX:ETS	2	0.100	0.905	1	0.011	0.918	2	0.011	0.918				COX:ETS	3	1.515	0.232			
	CS:LDH	2	0.386	0.682	1	7.181	**0.010**	2	1.751	0.185				CS:LDH	3	2.442	0.082			
	COX:LDH	2	0.495	0.613	1	0.812	0.373	2	0.156	0.856				COX:LDH	3	0.868	0.470			
	log_10_ CS:COX	2	1.431	0.250	1	1.057	0.310	2	1.223	0.304				log_10_ CS:COX	3	5.051	**0.014**			

Numbers in bold indicate significant *P*-values.

Design B: There was a significant effect of treatment on mean SMR (Table 3, Fig. 3). In particular, mean SMR values measured at the 10°C treatments were two times higher when compared to those at the 2°C treatments. However, mean SMR between the normoxic and hypoxic treatments were comparable regardless of the temperature.

#### Maximum metabolic rate

Mean MMR measured in northern shrimp exposed to elevated temperature, low pH and low oxygen in isolation and in combination are summarized in Table 2.

Design A: An increase in temperature led to an increase in mean MMR (Table 3, Fig. 3). In more detail, mean MMR values measured at the two highest temperatures tested were significantly higher (34 and 42%) than that reported at the lowest temperature condition, but comparable with each other. In addition, a significant decrease of 14% in mean MMR was observed at low pH (Table 3, Fig. 3). The interaction between temperature and pH was found to have no significant effect on mean MMR (Table 3).

Design B: There was a significant effect of treatment on mean MMR (Table 3, Fig. 3). Specifically, the two highest mean MMR values were observed in normoxic treatments, and conversely, the two lowest values in hypoxic treatments. The decrease in MMR caused by hypoxia was 55 and 41% at 2 and 10°C, respectively.

#### Aerobic scope

Mean AS measured in the northern shrimp exposed to elevated temperature, low pH and low oxygen in isolation and in combination are summarized in Table 2.

**Figure 4:**
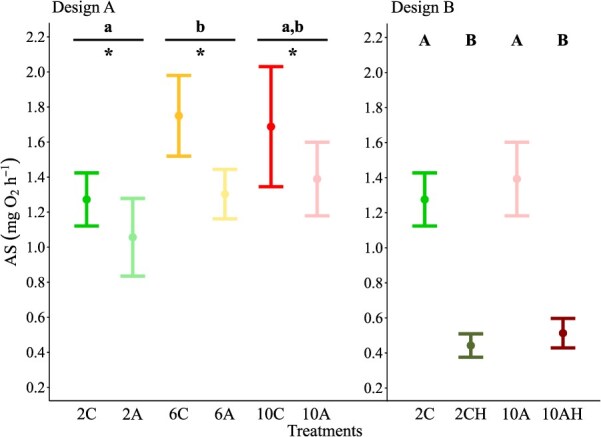
The effects of 30 d exposure to elevated temperature, low pH and low oxygen, in isolation and combination (Designs A and B), on mean AS of female shrimp *P. borealis*. Dots represent the mean value and the associated error bars the 95% CI. Lower case letters identify significant differences (*P* < 0.05) among temperature treatments independently of pH, whilst asterisks identify significant differences (*P* < 0.05) between pH treatments, combining all temperatures. Uppercase letters indicate significant differences (*P* < 0.05) among treatments.

Design A: Exposure to low seawater pH caused a significant decrease (20%) in mean AS at all temperatures combined (Table 3, Fig. 4). In addition, mean AS was significantly influenced by temperature (Table 3, Fig. 4). In more detail, a significant 31% increase in mean AS was observed between 2 and 6°C, whilst the mean AS measured at 10°C was found to be comparable to the mean AS obtained at the two lower temperatures tested. However, the interaction between temperature and pH did not exert a significant effect on mean AS (Table 3).

Design B: There was a significant effect of treatment on mean AS (Table 3, Fig. 4). Precisely, mean AS values measured in hypoxic treatments at both temperatures were significantly lower when compared to those measured in normoxic treatments at both temperatures (65 and 63%, 2 and 10°C, respectively). In addition, mean AS was comparable for the two treatments at the same DO level both in normoxic and hypoxic treatments.

#### Temperature coefficient

Design A only: Temperature coefficient (*Q*_10_) calculated for SMR and MMR between 2 and 10°C at both pH conditions varied between 1.02 and 2.15 at current pH and between 1.32 and 5.24 at low pH (Table 4). For both SMR and MMR*, Q*_10_ values calculated at current pH were higher for the 2 to 6°C interval, followed by the 2 to 10°C interval and then by the 6 to 10°C interval. Additionally, *Q*_10_ values calculated at low pH were in general higher that those calculated at current pH.

**Table 4:** Summary of temperature coefficient (Q_10_) values calculated for SMR and MMR of female shrimp *P. borealis* at current and low pH and experimental range of temperatures (2–10°C)

	pH	Temperature range		
		2–6°C	6–10°C	2–10°C
*Q*_10_ SMR	7.75	2.15	1.48	1.79
	7.40	2.99	1.74	2.28
*Q*_10_ MMR	7.75	2.15	1.02	1.49
	7.40	1.92	1.32	2.16

#### Enzymes activities and ratios

**Figure 5:**
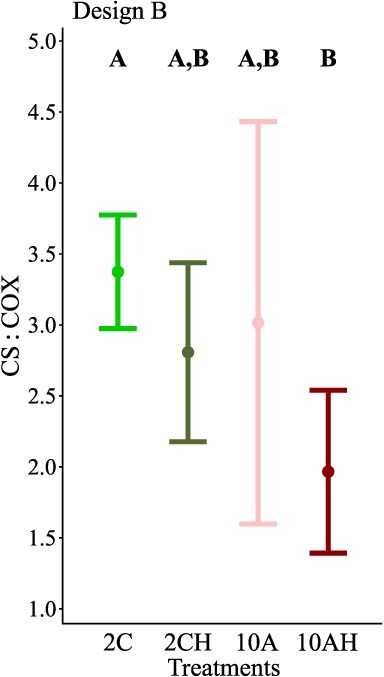
The effects of 30 d exposure to elevated temperature, low pH and low oxygen combined (Design B) on mean citrate synthase—cytochrome C oxidase ratio (CS:COX) specific activity in the muscle of female shrimp *P. borealis*. Dots represent the mean values and the associated error bars the 95% CI. Upper case letters indicate significant differences (*P* < 0.05) among treatments.

Mean specific enzymatic activities and their ratios measured in the abdominal muscle tissue of the northern shrimp exposed to elevated temperature, low pH and low oxygen in isolation and in combination (Designs A and B) are summarized in Table 2. The factors tested for both designs were found not to be significant on the mean activity of COX, ETS and LDH, nor on their ratios CS:ETS, COX:ETS and COX:LDH (Table 3). Exposure to low seawater pH (Design A) caused a significant decrease (20%) in mean CS activity (from 6.387 to 5.126 in current and low pH treatments, respectively, Table 3) and in mean CS:LDH (from 0.019 to 0.015 in current and low pH treatments, respectively, Table 3) when combining all temperatures tested. Additionally, mean CS:COX (Design B) for the 2C treatment was the highest (3.37) when compared to all other treatments and showed comparable means with the 2CH and 10A treatments (Table 3, Fig. 5). Finally, the mean CS:COX values reported at 2CH and 10A treatments were shown to be also comparable to the mean at the 10AH treatment (Fig. 5).

## Discussion

Altogether, our results raise concerns about the future conservation of the northern shrimp *P. borealis* in the Estuary and Gulf of St. Lawrence, from an ecological and socio-economic point of view. Ocean warming (OW), acidification (OA) and hypoxia in isolation are shown to control and limit shrimp aerobic performance. Despite the lack of interactive effects between temperature and pH in this study, we show that combined ocean global change drivers (OW + OA + hypoxia) severely affect the response of shrimp from the whole-organism to the cellular level. In particular, survival, metabolic rate and ultimately aerobic performance are severely reduced under combined ocean global change drivers. Nonetheless, shrimp cellular aerobic capacity, as expressed by enzymatic activity, is overall stable across all treatment combinations, with only a few exceptions. Interestingly, in shrimp exposed to combined ocean global changes, we observe an adjustment of the mitochondrial organization, possibly a plastic response, to maintain cellular energetic capacity. In general, our study confirms the importance of conducting multi-driver and multi-trait experiments when investigating the ability of marine organisms to tolerate combined ocean global change drivers, underlining the importance of accounting for both controlling and limiting effects. These effects are caused by the environmental factors defined by Fry (1971) as those that govern metabolic rates “by their influence on the state of molecular activation of the components of the metabolic chain” (i.e. controlling factor, such as temperature) and “by restricting the supply or removal of the materials in the metabolic chain”: i.e. limiting factor (e.g. oxygen).

Most studies so far conducted on the responses of the northern shrimp to a changing ocean have focused on temperature as the driver, which defines the species’ sensitivity. Shrimp were shown to be sensitive to increases in this driver throughout their life cycle, with females shown to be more sensitive than males (Brillon *et al.,* 2005; Daoud *et al.,* 2007, 2010; Arnberg *et al.,* 2013; Dupont-Prinet *et al.,* 2013). As an expression of the positive relationship between temperature and metabolic rate (measured via oxygen consumption) *Q*_10_ values generally decrease with increasing temperatures. Thus, as expected, *Q*_10_ values for the northern shrimp in our study are greater between 2 and 6°C than between 6 and 10°C for both standard and maximum metabolic rates (SMR and MMR, respectively). This suggests that female shrimp are more metabolically sensitive to increases in temperature from lower to intermediate temperatures: 2 to 6°C in the present study, and 2 to 5°C in Daoud *et al.* (2007). However, our results also show that shrimp can tolerate temperatures up to 6°C, as their aerobic scope (AS) increased by 31% between 2 and 6°C, confirming that temperature acts as a controlling factor for *P. borealis* metabolic rate (Fry, 1971; Pörtner, 2010). The absence of an increase in AS between 6 and 10°C likely shows that our highest temperature treatment is close to the upper critical temperature for shrimp (Allen, 1959; Brillon *et al.,* 2005). However, the absence of a drop in survival at 10°C after 30 d of exposure, when temperature is the only driver, supports the idea that shrimp can tolerate temperature increases close to those predicted to occur within the context of the ongoing OW (IPCC, 2022). This finding is in line with that by Allen (1959), who stated that adult *P. borealis* can live and reproduce at temperatures as high as 11.1°C.

Whilst being sensitive to low pH (*Q*_10_ values are higher in shrimp exposed to pH 7.40), our study confirms that adult shrimp can tolerate OA conditions, when it is the only driver to which they are exposed (Hammer and Pedersen, 2013). Indeed, OA does not affect survival when in isolation after 30 d of exposure, and little effect on AS. Shrimp AS decreases by 20% from the high to the low pH treatment, driven by the marked decrease in MMR. The reason for shrimp’ MMR decline at low pH is likely linked to the drop in extracellular pH, which decreases the affinity between respiratory pigments and oxygen, thus less oxygen is delivered to tissues (Pörtner *et al.,* 2004; Whiteley, 2011). When exposed to OA in isolation, the aerobic metabolism of shrimp provides less energy to support the different physiological functions (i.e. digestion, locomotion, growth and reproduction) requiring an adjustment in energy allocation, which can ultimately have short- and long-term negative effects at different ecological levels (Sokolova *et al.,* 2012).

Fry (1971) showed that hypoxia acts as a limiting factor for metabolism: organisms must decrease their oxygen uptake when dissolved oxygen levels in their environment are too low. The northern shrimp is able to maintain its SMR when exposed to acute severe hypoxia (~16% O_2_ sat. at 5°C and ~ 22% O_2_ sat. at 8°C; Dupont-Prinet *et al.,* 2013) and to chronic hypoxia (35% O_2_ sat. for 30 d, this study). However, both our study and that of Dupont-Prinet and colleagues show that adult shrimp MMR is severely reduced under hypoxic conditions. It decreases approximately by half, independently of temperature, due to the limiting effect that hypoxia exerts on MMR (Fry, 1971). In consequence, as SMR is maintained and MMR decreases, AS decreases by about 60% from normoxic to hypoxic conditions when shrimp are exposed for longer periods than first shown by Dupont-Prinet *et al.* (2013). Finally, and not surprisingly, survival is not affected by hypoxia in isolation because we chose a chronic, non-lethal, level of dissolved oxygen (35% O_2_ sat.) (Dupont-Prinet *et al.,* 2013).

While shrimp seem to be able to tolerate temperatures and pH predicted to occur for the end of the century (Lavoie *et al.,* 2020; IPCC, 2022) when tested in isolation, the co-occurrence of these environmental changes can lead to drastically different impacts. This confirms that multiple major drivers should always be studied together, in order to accurately determine the physiological responses of organisms to ocean changes. Indeed, shrimp survival is negatively affected by the combination of OW and OA (Dupont *et al.,* 2014) and the superimposed effect of hypoxia (Chemel *et al.,* 2020). In our study, survival is reduced by approximately 23% when shrimp are exposed to the combination of elevated temperature and low pH, and by approximately 58% when they are exposed to combined elevated temperature, low pH and low oxygen levels. Considering the hypoxia level we used (35% O_2_ sat.), our survival results can therefore be considered optimistic when compared to the predicted future conditions for 2100 for the EGSL. Indeed, average deep-water (300 m) oxygen level in 2020 was already as low as 15% O_2_ sat. in the SLE and less than 40% O_2_ sat. in the GSL (Blais *et al.,* 2021).

It is indeed interesting to compare the favourable (2C) *versus* combined scenario (10AH) to determine, from a conservation point of view, the magnitude of change in the physiological status of the northern shrimp under combined future global change drivers. Our results suggest that under future conditions maintenance costs will be almost two times higher (SMR increases by approximately 90%) and therefore shrimp will need to eat almost twice as much just to meet their maintenance costs. Additionally, MMR decreases by approximately 26%, meaning that the rate at which oxygen can be transported to the mitochondria for energetically demanding activities will decrease by a third. Consequently, AS decreases by approximately 60%, thus, the energy available to meet the aerobic metabolic demand in excess of SMR, but essential for survival, is reduced by more than half. Ultimately, the energy budget available to support physiological performance of shrimp, and ultimately fitness-related activities such as swimming, predatory and anti-predatory behaviour, and digestion, is greatly reduced. This in turn, is likely to have consequences on shrimp’s growth, body size and reproduction (Fry, 1947; Claireaux and Lefrançois, 2007; Pörtner and Knust, 2007), and ultimately their distribution and abundance (Pörtner and Knust, 2007; Cucco *et al.,* 2012).

However, the level of tolerance of a species can differ depending on the organizational level considered (Harvey *et al.,* 2014). In line with this, we show that exposure of female shrimp to isolated and combined ocean global change drivers induces little or no variation in aerobic and anaerobic enzyme activity, as measured in the abdomen muscle tissue. According to the often-observed aerobic compensation at low temperatures in ectotherms (Seebacher *et al.,* 2010), we would have expected higher CS or COX activity levels at the lower temperature and higher LDH activity at the higher temperature. However, most of these compensatory responses have been observed in fish, but recent studies on invertebrates have shown that these acclimation responses are not pervasive (Crockett *et al.,* 2001; Havird *et al.,* 2020; Hoffschröer *et al.,* 2024), showing that metabolic plasticity might not be a shared trait among ectotherms taxa. In this study, the specific activity of CS and the CS:LDH are only reduced by pH, both by approximately 20%. The activity levels and ratios for all other enzymes remain stable, suggesting that the northern shrimp can partly maintain its metabolic apparatus even when exposed to multiple global ocean change drivers, and for longer periods than previously shown by Dupont-Prinet *et al.* (2013) and Pillet *et al.* (2016). Nonetheless, these results also suggest that shrimp possess low ability for metabolic reorganization, as an increase in cellular aerobic capacity could have led to a compensation of the negative effects of combined drivers on the AS at the whole-organism level.

Interestingly, exposure to the combined scenario (OW + OA + hypoxia) decreases the CS:COX by approximately 41%. Specifically, CS is an enzyme located in the mitochondrial matrix, and its level of activity correlates to the mitochondrial volume (Moyes *et al.,* 1997), whilst COX is an integral membrane enzyme and its activity correlates with the mitochondrial membrane surface. The observed change might help to maintain aerobic efficiency, or capacity under combined ocean global change drivers, and could result from adjustments in the shape of mitochondria, specifically showing an increase in mitochondrial membrane surface (Heine *et al.,* 2023). Since COX is the last acceptor of electrons of the ETS and is responsible for the reduction of molecular oxygen (Heine *et al.,* 2023), higher COX over CS (and lower CS:COX) could therefore result in mitochondria with higher oxidative capacity relative to the reducing capacity (CS and Krebs cycle pathway), which might accommodate elevated aerobic capacity at high temperatures and lower pH and oxygen levels. In other words, mitochondria with relatively more COX could partly maintain oxidative phosphorylation capacity despite the negative effect of combined drivers at the whole-organism level. This would have to be validated by exploring the functional properties of these mitochondria at different environmental conditions.

Our results suggest that under the combination of ocean global change drivers shrimp cellular aerobic capacity is stable, whilst whole-organism physiological performance decreases in individuals surviving exposure, in addition to survival being severely reduced. These findings are overall in line with our hypothesis, and confirm the importance of integrating responses of a species at different levels of biological organization (Harvey *et al.,* 2014) to enhance its conservation (Wikelski and Cooke, 2006; Cooke *et al.,* 2013).

In conclusion, our results raise concerns about the future health of northern shrimp and its conservation status in the ESGL, considering the predicted changes in temperature, pH and dissolved oxygen for the end of the century and its limited adaptive potential (Leung *et al.,* 2023). Specifically, shrimp inhabiting the GSL are still most abundant at a depth of ~250 m, where pH is approximately 7.75 and where the temperatures they encounter went from ~5–6 to ~6–7°C, from 2006 to 2021 (Lavoie *et al.,* 2020; DFO, 2022). In the same period and at the same depth, DO levels went from ~35 to ~30% O_2_ sat. (DFO, 2022). At those depths, shrimp are likely to experience environmental conditions predicted to occur by the end of the century (IPCC, 2022) and tested in our study, or even worse as discussed above, supporting our concerns. Indeed, environmental changes are considered to have been responsible for part of the northern shrimp biomass loss in the southern regions of the northwest Atlantic during the last 15 y (e.g. Estuary and Gulf of St. Lawrence; DFO, 2020) as previously suggested for the shrimp stock collapse in the Gulf of Maine (Richards and Hunter, 2021). Our results suggest that shrimp abundance may decline to the point of possibly observing a local commercial extinction: for example, as already observed in the Gulf of Maine and in the western North Sea (ICES, 2004, 2013). This would severely impact the local fishing industry, leading to a loss of economic revenue, as the northern shrimp is the third most lucrative fishery in Eastern Canada (DFO, 2021).

Shrimp in the EGSL could mitigate the impact of simultaneous changes in temperature, pH and oxygen by migrating into more suitable habitats. Adult shrimp probably have limited capacities to migrate to other regions, due to their low mobility and the presence of physical barriers linked to the topography of the EGSL. However, they have been shown to be able to migrate bathymetrically to bottoms located at lower depths (<150 m) in the SLE, where environmental conditions are more favourable (DFO, 2022). Interestingly, shrimp’s vertical distribution change observed in the SLE is possibly a response to a drop in dissolved oxygen levels in deeper waters, as recent oxygen levels are close to or even below their critical oxygen level (Dupont-Prinet *et al.,* 2013). Indeed, hypoxia seems to greatly influence shrimp distribution, as in the SLE, where deep waters are most severely hypoxic, they occupy shallower waters than neighbouring GSL populations. In addition, shallower waters are colder and have higher pH (Mucci *et al.,* 2011, 2018; DFO, 2022). Colder temperatures combined with higher oxygen and pH levels represent more favourable conditions for the physiological status of the northern shrimp, but this strategy is only applicable if the sediment type and food availability match shrimp requirements. In this sense, suitable shallower habitats may be limited in terms of space, forcing shrimp to live at higher densities, which in turn will expose them, for example, to higher fishing and predator pressures. Whilst shrimp fishing quotas have been reduced greatly over recent years, potentially reducing pressure on these stocks, interestingly, *P. borealis* still makes up a large proportion in the diets of redfish and Greenland halibut despite shrimp biomass decreasing over the last decade (Brown-Vuillemin *et al.,* 2022). For these reasons, shrimps of the SLE are to be considered at risk of local commercial extinction. Our results will have to be considered together with a risk equivalent approach (Roux *et al.,* 2022) and integrated into models to help in predicting species’ future size, abundance, and distribution (see for example Tai *et al.* (2021)) to provide valuable scientific advice for fisheries management in a changing ocean and to assist in the maintenance of the northern shrimp’s health and conservation status in the ESGL.

## Supplementary Material

Web_Material_coaf076

## Data Availability

The original data generated in the course of the study and underlying this article are available on PANGAEA® Data Publisher at https://doi.org/10.1594/PANGAEA.988046.
