# Supplementary material for: Survival and aerobic performance of the northern shrimp are threatened by exposure to combined ocean global change drivers
Source: Conserv Physiol. 2025 Dec 24;13(1):coaf076. doi: 10.1093/conphys/coaf076 (PMC12728293; doi:10.1093/conphys/coaf076)
Supplement: Web_Material_coaf076 [file web_material_coaf076.zip › Guscelli_et_al._pandalus_Cons_Phys_Supp_Mat_clean.pdf]

Supplementary material

Survival and aerobic capacity of the northern shrimp are threatened by exposure to combined ocean global change drivers

Determination of the shortest reliable interval to estimate maximum metabolic rate (MMR)

To reduce the risk of underestimating MMR, flushes were cancelled for each group of five respirometers hooked to the same flush pump until the slope of all five shrimp was visibly reduced. This long closed phase lasted 56.7 min ± 23.9 (mean and SD). DO declines rapidly during this phase and further, the rate of decline diminishes rapidly. Another way of reducing the risk of underestimating MMR is to use rolling regressions (Zhang *et al.*, 2019). Contrary to sequential regressions, when one interval begins after the end of the previous interval, in rolling regressions each interval begins one point (1 s) after the beginning of the previous interval.

Using a too long interval to calculate the slope of DO decline over time could result in underestimating MMR. However, using a too short interval, especially with rolling regressions, could overestimate MMR if the DO decline is not perfectly smooth and is influenced by noise. The shortest reliable interval or window width (WW) was determined according to the methodology outlined by Zhang et al. (2019), with a few modifications. The objective is to use periods of DO declines and analyse them several times with sequential regressions, using a specific WW each time. The variability, either standard deviations (SDs) or coefficient of variations (CVs) of different WWs are then compared statistically.

In our study, the last 8.25 min of the long closed phase were used to determine the shortest reliable WW. The rate of DO decline changed slowly near the end of the long closed phases and it was possible to compare different WWs using these data, smoothed with a 15-point moving average to

reduce noise without changing the slope of the signal (Chabot *et al.*, 2021). For a given shrimp we calculated as many sequential regressions of DO over time as it was possible to fit into 8.25 min, investigating WWs from 0.5 to 4 min, in steps of 0.25 min. Thus, for example, 16 regressions were calculated for WW of 0.5 min and 2 regressions were calculated for WWs of 3–4 min. For each shrimp and WW, mean slope was calculated across all the regressions obtained, along with the SD and CV of the mean slopes, mean R^2 and the SD of the mean R^2. This was done for each of the 71 shrimp that were retained for statistical analyses. The final data set included 1065 observations (71 shrimp x 15 WWs) and the following variables: shrimp ID, WW, mean slope, slope SD, slope CV, mean R^2, R^2 SD.

Mean slopes were very similar for all WWs. However, SDs and CVs of the slopes, as well as mean R^2s and their SDs differed markedly. SDs were compared across WWs in R (R Core Team, 2020) using a mixed-effect model (package NLME, Pinheiro *et al.*, 2020), where shrimp ID was set as random variable. SDs were log transformed to improve the behaviour of the residuals. *Post-hoc* comparisons consisted in paired-t-tests (i.e., repeated measures for each shrimp, 2 WWs at a time) of all other WWs against the longest WW, 4 min, which was considered as most reliable. The risk of type I error was maintained at 0.05 using the Holm method (pairewise_t_test function of package rstatix, Kassambara, 2021).

Mean SDs were significantly greater than the control (WW = 240 s) when WW was less than 180 s (3 min) (Figure S 1) and 3 min was taken to be the shortest reliable WW to run rolling regressions covering the entire long closed phase. This analysis was repeated with log-transformed CVs, and the shortest reliable WW was again 3 min.

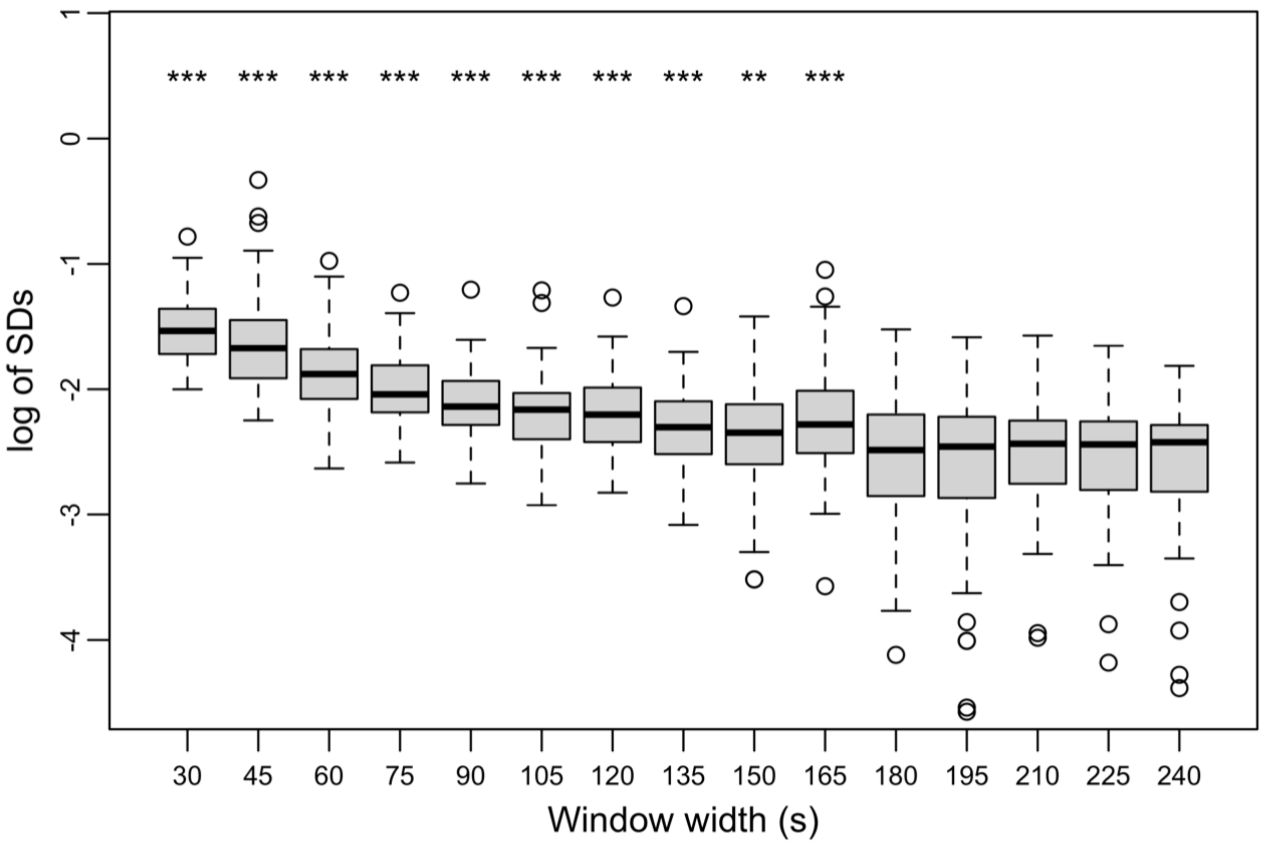

Figure S 1. Boxplot of SDs calculated on the slopes obtained for each window width of each shrimp. Asterisks (*, ** and *** correspond to $p < 0.05$, 0.01 and 0.001, respectively) show which WWs differ significantly from WW of 240 s.

Differences with the methodology of Zhang et al. (2019) were the choice of data type (end of long closed phases instead of blank respirometry cycles), their duration (8.25 instead of 20 min), the number of samples (71 instead of 5) and the use of a mixed-effects model considering that all WWs are analysed for each shrimp and are not independent. This also influenced *post-hoc* comparisons, which were made on the differences between pairs of WWs instead of on values of the two WWs directly, and the number of comparisons, which was reduced to comparisons of other WWs against a control WW (240 s or 4 min).

Even with log transformation, the assumptions of normality (Shapiro-Wilk test on the residuals of the model) and homoscedasticity (Levene's test using the median) were not met. However, as Anova-

type analyses are robust against departures from these assumptions when sample sizes are equal (Quinn and Keough, 2002) this analysis was judged adequate to select minimum window width. With a window width of 3 min, two slopes were measured for each shrimp in the tests described here, and two measures of R^2 were available for each shrimp. These were averaged, and the mean of these averages was 0.93, confirming that reliable estimates of the slope can be calculated with 3-min windows.

Table S1

Checklist of 53 essential criteria for the reporting of methods for aquatic intermittent-flow respirometry (Killen *et al.*, 2021).

**Number**	**Criterion and Category**	**Response**	**Value (where required)**	**Units**
	**EQUIPMENT, MATERIALS, AND SETUP**			
**1**	Body mass of animals at time of respirometry	Wet mass was measured immediately after respirometry.		
**2**	Volume of empty respirometers	Including recirculation loop.	≈ 378 ± 19.9	mL
**3**	How chamber mixing was achieved	With a recirculation loop, consisting of a recirculating pump (AD20P-1230C, DollaTek, Hong Kong, China) and gas tight Tygon ® tubing.		
**4**	Ratio of net respirometer volume (plus any associated tubing in mixing circuit) to animal body mass		40.6 ± 6.8	
**5**	Material of tubing used in any mixing circuit	Gas tight Tygon ® tubing.		
**6**	Volume of tubing in any mixing circuit		4.3	mL
**7**	Confirm volume of tubing in any mixing circuit was included in calculations of oxygen uptake	Yes.		
**8**	Material of respirometer (e.g. glass, acrylic, etc.)	Glass.		
**9**	Type of oxygen probe and data recording	Fiber optic oxygen probes (OXYPro DP-PSt3, PreSens Precision Sensing GmbH) coupled to an oxygen meter (Oxy-4 mini or Fibox 3, PreSens Precision Sensing GmbH, Regensburg, Germany).		
**10**	Sampling frequency of water dissolved oxygen		1	Measurement s^{-1}
**11**	Describe placement of oxygen probe (in mixing circuit or directly in chamber)	The oxygen probe was placed on the positive pressure side of the recirculation loop.		
**12**	Flow rate during flushing and recirculation, or confirm that chamber returned to normoxia during flushing	Chamber returned to normoxia during flushing.		
**13**	Timing of flush/closed cycles	Respirometers were flushed with experimental treatment seawater for 300 s and closed for 660 s.		

**14**	Wait (delay) time excluded from closed measurement cycles		180	s
**15**	Frequency and method of probe calibration (for both 0 and 100% calibrations)	Probes were calibrated before the beginning of each respirometry measurement. For the 0% calibration we prepared a solution of sodium sulphite and boric acid in freshwater. Probes were calibrated in the solution at the experimental temperature once the probe signal was stable. For the 100% calibration, probes were calibrated in constantly aerated seawater in the experimental tank at the same temperature as for the 0% calibration.		
**16**	State whether software temperature compensation was used during recording of water oxygen concentration	No, a constant temperature was assumed, but these tanks were known to have stable temperature (± 0.1 °C).		
	**MEASUREMENT CONDITIONS**			
**17**	Temperature during respirometry	According to the treatment of measurement.	2, 6 or 10	°C
**18**	How temperature was controlled	With a feedback system (1/16 DIN Micromega autotune PID Temperature, Omega Engineering inc., Norwalk, USA) that regulated the automatic mixing of cold and hot water to provide each tank with sea water at the set temperature.		
**19**	Photoperiod during respirometry	24h dark (respirometry tanks were completely surrounded by a thick opaque curtain).		
**20**	If (and how) ambient water bath was cleaned and aerated during measurement of oxygen uptake (e.g. filtration, periodic or continuous water changes)	We used water flow-through tanks supplied with sea water from two reservoirs at a constant flow rate of 3.5 L min^{-1} and equipped with a submersible pump (1048, Eheim, Stuttgart, Germany) that allowed water mixing.		
**21**	Total volume of ambient water bath and any associated reservoirs	Tank water volume = 240 L and reservoirs volume = 750 L each for cold and warm water.		
**22**	Minimum water oxygen dissolved oxygen reached during closed phases.	For MMR, we used for each shrimp the lowest DO value observed at the end of the long closed phase following chase, even though MMR (the steepest part of the slope) was observed at much higher		% sat.

		DO. For SMR, we used for each shrimp the average of minimum DO at the end of all closed phases used to estimate SMR.<br><br>Normoxia (n = 53 shrimp)<br>MMR (mean ± SD):<br>SMR (mean ± SD):<br><br>Hypoxia (n = 18 shrimp)<br>MMR (mean ± SD):<br>SMR (mean ± SD):	 <br><br><br><br>68 ± 10<br>94 ± 3<br><br><br>21 ± 3<br>27 ± 3	
**23**	State whether chambers were visually shielded from external disturbance	Yes.		
**24**	How many animals were measured during a given respirometry trial (i.e. how many animals were in the same water bath)		10	
**25**	If multiple animals were measured simultaneously, state whether they were able to see each other during measurements	Ten respirometers were side by side in a tank, in darkness, but it is still possible that there was enough light in daytime for shrimp to see neighbouring respirometers.		
**26**	Duration of animal fasting before placement in respirometer	5 days fast was selected to ensure that the digestive tract was empty even at 2 °C. Fasting period was kept constant at the other temperatures.	5	days
**27**	Duration of all trials combined (number of days to measure all animals in the study)	Respirometry trials started on the 6th of august 2018 and ended on the 20th of august 2018. The respirometry trials combined lasted a total of 15 d.		
**28**	Acclimation time to the laboratory (or time since capture for field studies) before respirometry measurements	Approximately 8 weeks before the beginning of the experiment that itself lasted 30 days.		
	**BACKGROUND RESPIRATION**			
**29**	State whether background microbial respiration was measured and accounted for, and if so, method used (e.g. parallel measures with empty respirometry chamber, measurements before and after for all chambers while empty, both)	Background $\dot{M}O_2$ was measured and a linear regression was fitted to the background respiration values to estimate the evolution of background respiration with time. This regression of background respiration as a function of time for each shrimp was used to correct $\dot{M}O_2$. It should be noted that background respiration was in fact a spurious increase in DO caused by changes in pressure in the respirometers caused by the recirculation		

		pumps; these changes were repeatable and treated as if they were background respiration, i.e., background slope were subtracted from respiration slopes, even though they were of the opposite sign.		
**30**	State if background respiration was measured at beginning and/or end, state how many slopes and for what duration	Background $\dot{M}O_2$ was measured before shrimp were introduced into the respirometers and after shrimp removal for a minimum of 4 h and 15 slopes each.		
**31**	State how changes in background respiration were modelled over time (e.g. linear, exponential, parallel measures)	Linear regression, with slopes close to zero in the vast majority of cases. The slope was always close to zero, indicating that background respiration was negligible and that most of the detected change in DO during each cycle was caused by pressure changes.		
**32**	Level of background respiration (e.g. as a percentage of SMR)	mean ± SD	13.9 ± 9.0	% of SMR
**33**	Method and frequency of system cleaning (e.g. system bleached between each trial, UV lamp)	The system was cleaned with freshwater before the beginning of each respirometry trial. Additionally, each respirometer was gently rubbed with a brush.		
	**STANDARD OR ROUTINE METABOLIC RATE**			
**34**	Acclimation time after transfer to chamber, or alternatively, time to reach beginning of metabolic rate measurements after introduction to chamber	Time to reach beginning of metabolic rate measurements after introduction to chamber.	15 and 24 for normoxic and hypoxic treatments respectively	h
**35**	Time period, within a trial, over which oxygen uptake was measured (e.g. number of hours)		48	h
**36**	Value taken as SMR/RMR (e.g. quantile, mean of lowest 10 percent, mean of all values)	Quantile method with a q of 0.2.		
**37**	Total number of slopes measured and used to derive metabolic rate (e.g. how much data were used to calculate quantiles)	mean ± SD (excluding slopes rejected because of low R^2, line 40)	105 ± 17	
**38**	Whether any time periods were removed from calculations of SMR/RMR (e.g. data during acclimation, periods of high activity [e.g. daytime])	Recovery and acclimation periods of 15 h and 24 h for normoxic and hypoxic treatments respectively.		
**39**	r^2 threshold for slopes used for SMR/RMR (or mean)	For most shrimp, minimum r^2 was set to 0.95 or between 0.9 and 0.94 (n = 52 and 17, respectively). For two shrimp, r^2 was decreased to 0.87 and 0.80. MMR was calculated by rolling regression without a		

		minimum r^2, but average r^2 was 0.99 for the steepest part of the slope.		
**40**	Proportion of data removed due to being outliers below r-squared threshold	mean ± SD	2.36 ±3.2	%
	**MAXIMUM METABOLIC RATE**			
**41**	When MMR was measured in relation to SMR (i.e. before or after)	MMR was measured before SMR.		
**42**	Method used (e.g. critical swimming speed respirometry, swim to exhaustion in swim tunnel, or chase to exhaustion)	Individuals were chased to exhaustion, defined as the moment they were not able to flick their tail anymore.		
**43**	Value taken as MMR (e.g. the highest rate of oxygen uptake value after transfer, average of highest values)	We retained the steepest slope over the long closed phase following introduction of each shrimp in the respirometer, or the highest $\dot{M}O_2$ observed during the regular intermittent-flow cycles, due to spontaneous activity. MMR was the highest of the two values and in the majority of cases, it was the post-chase value.		
**44**	If MMR measured post-exhaustion, length of activity challenge or chase (e.g. 2 min, until exhaustion, etc.)	Chased until no longer responsive to pinching of tail. Note that shrimp were then exposed to air for 1 additional minute. mean ± SD	4.45 ± 0.12	min
**45**	If MMR measured post-exhaustion, state whether further air-exposure was added after exercise	Yes, immediately after being chased to exhaustion, shrimp were exposed for 1 min to air to further increase their oxygen debt.		
**46**	If MMR measured post-exhaustion, time until transfer to chamber after exhaustion or time to start of oxygen uptake recording	After the 1 min air exposure, shrimp were rapidly but carefully transferred into individual respirometers.		
**47**	Duration of slopes used to calculate MMR (e.g. 1 min, 5 min, etc.)	See “Determination of the shortest reliable interval to estimate maximum metabolic rate (MMR)“ for a justification of this duration.	3	min
**48**	Slope estimation method for MMR (e.g. rolling regression, sequential discrete time frames)	Rolling regression.		
**49**	How absolute aerobic scope and/or factorial aerobic scope is calculated (i.e. using raw SMR and MMR, allometrically mass-adjusted SMR and MMR, or allometrically mass-adjusting aerobic scope itself)	Mass-specific MMR – Mass-specific SMR.		

	**DATA HANDLING AND STATISTICS**			
**50**	Sample size		80	individuals
**51**	How oxygen uptake rates were calculated (software or script, equation, units, etc.)	Our own scripts to smooth raw data, plot and calculate individual slopes, correct for blank respiration and calculate $\dot{M}O_2$. fishMO2 R package for calculation of SMR. Our own scripts to calculate MMR with rolling regressions.		
**52**	Confirm that volume (mass) of animal was subtracted from respirometer volume when calculating oxygen uptake rates	Yes.		
**53**	State whether analyses accounted for variation in body mass and describe any allometric mass-corrections or adjustments	Yes, we used mass specific metabolic traits, which were still related to shrimp mass. For this reason, shrimp mass was used as a covariable in our statistical analyses.		

4

References

Chabot D, Zhang Y, Farrell AP (2021) Valid oxygen uptake measurements: using high r2 values with good intentions can bias upward the determination of standard metabolic rate. *J Fish Biol*. doi:10.1111/jfb.14650

Kassambara A (2021) Package ‘rstatix’. Pipe-Friendly Framework for Basic Statistical Tests. *https://CRANR-project.org/package=rstatix*.

Killen SS, Christensen EAF, Cortese D, Závorka L, Norin T, Cotgrove L, Crespel A, Munson A, Nati JJH, Papatheodoulou M, *et al.* (2021) Guidelines for reporting methods to estimate metabolic rates by aquatic intermittent-flow respirometry. *J Exp Biol* 224(18): jeb242522.

Pinheiro J, Bates D, DebRoy S, Sarkar D, R Core Team (2020) Nlme: Linear and Nonlinear Mixed Effects Models. *R Packag version 31-148, <URL https//CRANR-project.org/package=nlme>*.

Quinn GP, Keough MJ (2002) Experimental design and data analysis for biologists. Cambridge university press.

Zhang Y, Gilbert MJH, Farrell AP (2019) Finding the peak of dynamic oxygen uptake during fatiguing exercise in fish. *J Exp Biol* 222. doi:10.1242/jeb.196568
